# Selection and Validation of Reliable Reference Genes for *Liquidambar formosana* Leaves with Different Leaf Colors

**DOI:** 10.3390/cimb46090560

**Published:** 2024-08-27

**Authors:** Fangwei Zhou, Liang Xu, Congguang Shi, Shaozong Yang, Yahui Chen

**Affiliations:** 1 Zhejiang Key Laboratory of Forest Genetics and Breeding, Zhejiang Academy of Forestry, Hangzhou 310023, China; zhoufangwei@njfu.edu.cn (F.Z.); jachary@163.com (L.X.); scgstone988@aliyun.com (C.S.); 2 Jiangsu Academy of Forestry, Nanjing 211153, China

**Keywords:** *Liquidambar formosana*, real-time quantitative PCR, reference gene

## Abstract

*Liquidambar formosana* Hance is renowned for its rich leaf color and possesses notable advantages, such as robust adaptability, strong resistance to diseases and pests, and rapid growth, making it a preferred choice for urban greening and carbon sequestration forest initiatives. The completion of whole-genome sequencing of *L. formosana* has spurred an increased interest in exploring the molecular mechanisms underlying seasonal changes in leaf color, marking a significant focus in *L. formosana* breeding research. However, there is currently a lack of stable reference genes suitable for analyzing the expression patterns of functional genes in *L. formosana* exhibiting varying leaf colors. This study selected five *L. formosana* varieties with significant differences in leaf colors. Through the RT-qPCR analysis, and evaluation using BestKeeper, geNorm, NormFinder, Delta Ct, and RefFinder, the expression stability of 14 candidate reference genes was examined. Consequently, two reference genes (*LifEF1-α* and *LifACT*) with stable expression, suitable for RT-qPCR of *L. formosana* with diverse leaf colors, were identified. The stability of these selected reference genes was further validated by examining the *LifbHLH137* gene, which promoted the biosynthesis of anthocyanins. This advancement facilitated molecular biology and genetic breeding investigations of *L. formosana*, providing essential data for the precise quantification of functional genes associated with leaf color variation.

## 1. Introduction

*Liquidambar formosana* Hance, classified under the family Hamamelidaceae and genus *Liquidambar*, is a tall deciduous tree esteemed as both a crucial afforestation species and an exceptional landscape ecological tree. Renowned for its rich leaf color, it predominantly thrives in southern and central China, alongside the tropical regions of Southeast Asia, with additional occurrences in the eastern United States, Mexico, and Guatemala [1]. *L. formosana* exhibits a tall tree shape, dense foliage, and robust adaptability, demonstrating resilience to drought and infertile soil conditions [2]. Concurrently, the escalating demand for ornamental flora and advancements in landscaping have propelled *L. formosana* into the spotlight because of its rich, bright color and high ornamental value, thereby establishing them as the preferred species for national greening initiatives [3].

Most autumn-colored leaf tree species are deciduous plants, mainly due to insufficient winter light and lower temperatures. The self-sufficiency cost of plants during the leaf-shedding period will exceed the capacity accumulation of photosynthesis. Therefore, plants choose to enter a brief dormancy period to reduce their own nutrient consumption [4]. Simultaneously, the accumulation of antioxidant compounds, such as anthocyanins, serves as a mechanism to cope with environmental changes. Notably, anthocyanins tend to accumulate significantly during the period when leaf color begins to change [5,6]. Chlorophyll degradation products exhibit antioxidant properties that can preserve cellular activity and mitigate aging processes [6,7]. Consequently, the degradation of chlorophyll and the synthesis of anthocyanins reflect the adaptive regulatory mechanisms of plants in response to environmental conditions. Investigating the molecular mechanisms underlying these processes holds substantial biological significance for the development of superior plant varieties through advanced scientific and technological methodologies.

Current research on *L. formosana* predominantly focuses on resource distribution, breeding of improved varieties, population genetics and evolution, and ecological monitoring [3,8]. However, most studies concerning the color changes of *L. formosana* leaves typically focus only on the phenotypic traits and lack in-depth exploration of the molecular regulatory mechanisms underlying these changes. Understanding the key regulatory factors for leaf color variation in *L. formosana* remains a significant research area considering the combined impact of genetic background and environmental factors on color variation. Research on the color change of *L. formosana* leaves primarily focuses on physiological and biochemical aspects, such as photosynthesis and pigment content [1,9], while the molecular mechanisms driving these changes still lack comprehensive genetic information, inhibiting the development and utilization of *L. formosana* leaf color resources. A definitive solution for generating new *L. formosana* germplasms with diverse leaf colors via biotechnology and gene editing remains elusive, and the molecular regulatory mechanisms governing leaf color variation remain unclear. The completion of whole-genome sequencing for *L. formosana* has significantly advanced molecular biology and genetic breeding research in this species [10]. The advancement of the *L. formosana* industry correlates with the progress of the agricultural and forestry economy and establishment of an ecological civilization. Therefore, there is an urgent need for comprehensive research into the seasonal fluctuations in *L. formosana* leaf color, the identification of key genes that dictate these color changes, and the examination of expression patterns and regulatory mechanisms of crucial functional genes affecting the diversity of *L. formosana* leaf color. This study can provide a foundation for *L. formosana* tree breeding.

Real-time quantitative PCR (RT-qPCR) is now widely used to study gene transcriptional expression levels due to its convenience, speed, specificity, and sensitivity. However, factors such as RNA and cDNA quality, sample dilution ratio, and experimental accuracy can affect the RT-qPCR accuracy [11]. Therefore, employing appropriate reference genes is crucial for standardized analysis of target gene expression levels [12]. The ideal reference genes are stably expressed in cells, typically encompassing housekeeping genes, such as actin, 18S Ribosomal RNA, tubulin, and ubiquitin, which maintain fundamental cellular activities [13,14]. Nevertheless, recent studies have indicated that variations in species and tissue organs can alter the transcription of housekeeping genes. The leaves from various *Zantedeschia hybrida* varieties were collected, and six commonly used reference genes (*18S rRNA*, *ACT*, *EF1-α*, *GAPDH*, *LEU*, and *TUB*) were assessed. The analysis that was conducted using the reference gene analysis methods and software revealed that *18S rRNA*, *LEU*, and *GAPDH* exhibited greater stability across different varieties, whereas *ACT*, *EF1-α*, and *18S rRNA* were the most stable across different tissues [15]. This suggested that optimal reference genes may vary across developmental stages and tissues. Moreover, the stability of reference genes can differ within the same plant under different environmental conditions. Hence, the selection of appropriate reference genes should be aligned with the specific experimental conditions to minimize experimental errors.

Currently, there is a scarcity of suitable reference genes for investigating functional genes linked to the *L. formosana* leaf color. Commonly used reference genes may present instability across different plants and experimental conditions, potentially affecting the accuracy of target gene identification. In this study, the leaves from five *L. formosana* varieties exhibiting distinct leaf color differences in September and December were used as experimental samples. Based on transcriptome data of *L. formosana*, 14 potential reference genes were identified. Various analyses, including fluorescence-quantitative PCR, amplification curve and melting curve analyses, LinRegPCR amplification efficiency analysis, and stability assessments using BestKeeper, geNorm, NormFinder, Delta Ct, and RefFinder, were conducted to identify two stable reference genes (*LifEF1-α* and *LifACT*) suitable for fluorescence-quantitative PCR. The stability of the selected reference genes was validated using *LifbHLH137*, which enhanced anthocyanin biosynthesis. This study addressed the gap in stable reference genes for studying *L. formosana* leaf color variation, advanced molecular biology, and genetic breeding research, and offered data support for the precise quantification of functional genes related to leaf color variation, thereby enhancing research stability and accuracy.

## 2. Materials and Methods

### 2.1. Plant Materials

**Figure 1:**
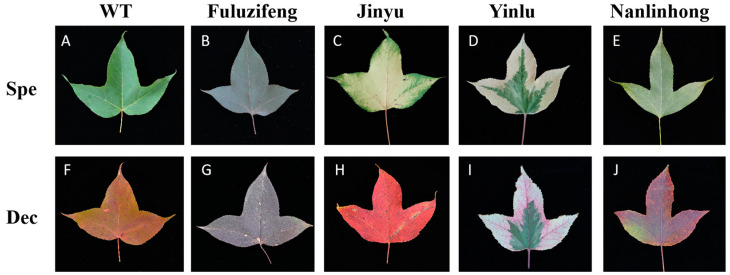
The leaves of *L. formosan* with different leaf colors, including leaves from September (Spe) and December (Dec) of WT, “Jinyu”, “Fuluzifeng”, “Yinlu”, and “Nanlinhong”. (**A**–**E**) WT, “Jinyu”, “Fuluzifeng”, “Yinlu”, and “Nanlinhong” leaves in September. (**F**–**J**) WT, “Jinyu”, “Fuluzifeng”, “Yinlu”, and “Nanlinhong” leaves in December.

For this study, experimental materials comprised the leaves from *L. formosana*, specifically, selected variants with distinct leaf colors: “Jinyu”, “Fuluzifeng”, “Yinlu”, and “Nanlinhong”. Three plants from each variant exhibiting consistent growth and height were randomly selected for sample collection. The complete leaves were harvested in September and December of 2023 (Figure 1). Three replicates were collected from each tree per period, which were promptly frozen in liquid nitrogen upon collection and stored at −80 °C until further applications.

### 2.2. Total RNA Extraction and cDNA First-Strand Synthesis

The total RNA was extracted from various leaf tissues using the RNAprep Pure Plant Plus Kit (TIANGEN, Nanjing, China). The integrity and concentration of the total RNA were assessed using 1.2% agarose gel electrophoresis and an ultra-micro spectrophotometer (Thermo Fisher, Waltham, MA, USA), respectively. After the extraction, the total RNA concentration was diluted to 100 ng/µL. The first strand of cDNA was synthesized using the Fastking gDNA Dispelling RT SuperMix (TIANGEN, Nanjing, China) and stored at −20 °C for subsequent experiments.

### 2.3. Primer Design and Specific Detection

From transcriptome sequencing data of *L. formosan* and common reference genes in plants, 14 candidate reference genes were selected: *LifEF1-α*, *LifACT*, *Lifα-TUB1*, *Lifβ-TUB*, *LifARF2*, *LifCDC2*, *LifDnaJ*, *LifGAPDH*, *LifH2A*, *LifHIS*, *LifLTP*, *LifSAND*, *LifUBC*, and *LifUBQ*. The primer design was conducted using Primer Premier 5.0, adhering to standards such as an annealing temperature of 54–62 °C, primer length of 18–25 bp, and amplification fragment length of 100–300 bp [16]. The primer specificity was confirmed using the BLAST tool, and the detailed sequences and product lengths are provided in Table 1. The primers were synthesized by Shenggong Biotechnology Co. Ltd. (Shanghai, China). The primer specificity was assessed by the PCR amplification and RT-qPCR melting curve analysis, using mixed cDNA from the leaves of five different *L. formosana* varieties as templates. The PCR reaction system comprised 25.0 µL, including 12.5 µL of 2× Taq Fast PCR Mix, 1.0 µL of each upstream and downstream primer at 10 µmol/L, 1.0 µL of template cDNA at 100 ng/µL, and RNase Free ddH_2_O supplemented to 25.0 µL.

### 2.4. Real-Time Fluorescence-Quantitative PCR of Candidate Reference Genes

The real-time fluorescence-quantitative PCR detection was conducted using the QIAquant 96 2plex (QIAGEN, Hilden, Germany) fluorescence-quantitative PCR system and PowerUp™ SYBR™ Green Master Mix (Applied Biosystems, Waltham, MA, USA), with cDNA from leaves of five different *L. formosana* varieties (“WT”, “Jinyu”, “Fuluzifeng”, “Yinlu”, and “Nanlinhong”) collected in September and December serving as templates. The reaction system comprised 10 μL of PowerUp™ SYBR™ Green Master Mix, 0.2 µL each of 10 µmol forward and reverse primers, 2.0 µL of cDNA templates at varying dilutions, and RNase-free ddH_2_O supplemented to a total volume of 20.0 µL. The amplification program included pre-denaturation at 95 °C for 3 min, followed by 40 cycles of 95 °C for 15 s, 60 °C for 15 s, and 72 °C for 30 s. After the amplification, the melting curve analysis was performed at 55–95 °C. All analyses were conducted in triplicates.

### 2.5. Establishment of Reference Gene Primer Standard Curves

Equal amounts of cDNA templates from all samples were combined and diluted with ddH_2_O to 5 concentrations, each of which was diluted 5 times (1, 1/5, 1/25, 1/125, and 1/625), to establish standard curves. The RT-qPCR amplification was performed using the QIAquant 96 2plex (QIAGEN, Germany) to obtain Ct values for each candidate reference gene at different template dilutions. The standard curve for each candidate reference gene was constructed by plotting the logarithmic relationship between the average Ct value and continuously diluted template cDNA. The slope (K) and correlation coefficient (R^2^) were derived from the standard curve, and the PCR amplification efficiency (E) was calculated using the formula: E = [5 ^(1/−K)^ − 1] × 100%, for each primer pair [17]. The comprehensive evaluation was conducted to ensure that the system (including primers and templates) and amplification program met the requirements for the RT-qPCR analysis. The criteria included a correlation coefficient R^2^ > 0.99 and an amplification efficiency E value between 90% and 120% [17]. All samples were subjected to three repetitions to ensure the reliability of the experimental data.

### 2.6. Data Analysis

(1) The geNorm version 2003 software, a fundamental programming tool, can assess reference gene stability via stability parameter M value calculation [18]. A threshold of M = 1.5 was established, where M < 1.5 indicated reference gene suitability, and lower M values indicated superior stability. Conversely, for M values exceeding 1.5, the increasing M correlated with the heightened instability of the reference gene. Consequently, this gene was unsuitable as a reference gene. In addition, when calculating multiple reference genes, the geNorm software can identify the optimal number of reference genes by assessing a new paired variation (V) value of the normalization factor for reference genes. By utilizing the ratio of V_n/n+1_, the software assessed the impact of introducing new internal reference genes on the standard factor. The default threshold for the V-value was set at 0.15. If V_n/n+1_ < 0.15, it indicated stability among the n internal reference genes for normalization. Conversely, if V_n/n+1_ > 0.15, the introduction of the n + 1st gene became necessary. This software reliably determined a suitable number of reference genes [19].

(2) The NormFinder version 0953 software normalized candidate genes based on their expression stability within a given sample group and experimental design. Its methodology closely paralleled the geNorm, involving the calculation of ΔCt values and their subsequent transformation into 2^−ΔCt^ values using Excel functions. These values represent the relative expression levels of candidate reference genes from which the stable expression value (S) was derived. The reference gene with the smallest stable value was deemed the most suitable. Compared to the geNorm, the NormFinder identified only one stable reference gene and did not determine the optimal number of reference genes. Nevertheless, it offers guidance for identifying the best reference gene [20].

(3) The BestKeeper version 2003 algorithm employed the repeated-pair correlation and regression analyses to conduct a correlation analysis for each reference gene. The original Ct values were directly imported into the BestKeeper Excel table using this algorithm. The coefficient of variation (CV) and standard deviation (SD) were used to comprehensively assess the stability of the reference gene. A lower CV ± SD value indicated greater stability of the reference gene. A comparison of these values determined the ranking of the final reference genes based on their stability [21].

(4) The ΔCt version 2003 algorithm discerns stable reference genes by comparing the relative expression levels of gene pairs within each sample. The consistent ΔCt values across various samples indicated stable gene expressions, whereas fluctuations indicated instability. By introducing additional genes for comparison and subsequent sorting, suitable reference genes can be selected based on the experimental criteria [22].

(5) The RefFinder version 2003 software systematically evaluated the stability of internal reference genes obtained from the geNorm version 2003, NormFinder version 0953, ΔCt version 2003, and BestKeeper version 2003 software by computing their geometric mean weights. Lower geometric means could indicate greater stability of the internal reference genes. The stability of all reference genes was comprehensively assessed through scoring, leading to the selection of the most stable internal reference genes [23].

### 2.7. Stability Verification of Reference Genes

*LifbHLH137*, known to promote anthocyanin biosynthesis, was selected to assess the stability of the selected reference genes [24]. The *LifEF1-α* and *LifACT*, identified as the most stable reference genes, were employed to analyze *LifbHLH137* expression in leaves of WT, “Jinyu”, “Fuluzifeng”, “Yinlu”, and “Nanlinhong”. The unstable expressions of *LifCDC2* and *LifLTP* genes served as the controls. The *LifbHLH137* expression was evaluated using the 2^−ΔΔCt^ method [25,26].

## 3. Results

### 3.1. RNA Quality Testing and Primer Specificity Validation

This study employed NanoDrop™ One (Thermo Fisher, Waltham, MA, USA) and 1% agarose gel to assess the total RNA mass extracted from leaves of WT, “Jinyu”, “Fuluzifeng”, “Yinlu”, and “Nanlinhong” in September and December (Figure 1). The RNA concentration ranged from 140 to 400 ng/μL, with the OD_260/280_ values between 2.0 and 2.3, indicating high purity without protein or phenol contamination. The agarose gel electrophoresis revealed clear main bands for each sample without dispersion or tailing. The brightness ratio of the 28S rRNA to 18S rRNA bands was approximately 2:1, indicating minimal RNA degradation and high integrity, meeting the subsequent testing requirements.

Based on the transcriptome data from *L. formosan*, 14 candidate reference genes with stable expression across various tissues were identified. The PCR amplification was conducted using equimolar amounts of mixed cDNA from the leaves of the five *L. formosan* varieties as templates. The results revealed single and intense target fragments devoid of primer dimerization or non-specific amplification. The fragment sizes ranged from 102 to 264 bp, which was consistent with expectations (Figure S1). The subsequent validation of primer specificity via the RT-qPCR revealed single dissolution peaks for each candidate reference gene free from hairpin structures or primer dimers (Figure S2). The overlap of amplification curves from repeated samples indicated appropriate annealing temperatures and excellent primer specificity for each reference gene (Figure S3). The standard curves were established using the cDNA templates with varying concentration gradients, demonstrating primer amplification efficiency (E) within an acceptable range of 90% to 120%, ranging from 99.31% to 117.49%. The linear correlation coefficients (R^2^) showed minimal differences, ranging from 0.9765 to 0.9961 (Table 1). These results confirmed the soundness of the primer design for the 14 candidate reference genes, ensuring favorable specificity and amplification efficiency for the RT-qPCR. They provided accurate and reliable data for reference gene stability assessments.

### 3.2. Reference Primer Amplification Ct Value Analysis

**Figure 2:**
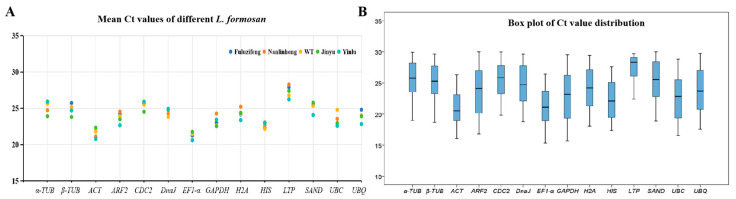
The distribution of Ct values of the 14 candidate reference genes in *L. formosan*. (**A**) Mean Ct values of reference genes in different *L. formosan*. (**B**) Box plot of Ct values for the 14 candidate reference genes. The median is depicted as a horizontal line, with the upper boundary of the box denoting the upper quartile, the lower boundary representing the lower quartile, and the outermost lines in each box corresponding to the maximum and minimum Ct values.

The Ct value, which is inversely related to gene expression, reflects the expression level across all experimental samples. The lower Ct values corresponded to the higher gene expression levels. The box plots of Ct values for each reference gene across different samples indicated a range of 15.69 to 29.97, indicating moderate expression abundance (Figure 2). The mean Ct values for the 14 reference genes ranged from 21.35 to 27.32. The *LifEF1-α* demonstrated the highest expression level across all samples (average Ct value: 21.35), whereas the *LifLTP* exhibited the lowest (average Ct value: 27.32). Greater gene stability correlated with a narrower range of Ct values. Preliminary observations from box plot analysis indicated notable variations in the expression levels of different reference genes in *L. formosan*. The Ct values of *LifGAPDH* presented the widest range among the 14 reference genes, spanning from 15.69 to 29.56, indicating the low expression stability. Conversely, the *LifACT* (16.05–26.32) and *LifEF1-α* (15.32–26.43) exhibited narrower Ct value ranges, suggesting a more stable gene expression (Figure 2). These findings indicated the varying expression levels of different reference genes in the leaves of different *L. formosan* varieties and developmental stages. Although most candidate reference genes displayed relatively high expression levels with minimal variation, ensuring their suitability as reference genes, the consistency of their expression levels across samples was less pronounced. Therefore, employing 5 statistical algorithms to evaluate the expression stability of the 14 reference genes across different colors of *L. formosan* leaves could be imperative for identifying the optimal reference genes for standardizing target gene expression.

### 3.3. Analysis of Gene Expression Stability

To assess the expression stability of the 14 candidate reference genes across various *L. formosan* leaf varieties, ΔCt, BestKeeper, geNorm, and NormFinder were employed for the statistical analysis of gene expression levels in diverse leaf tissues. The RefFinder was employed to rank the overall stability and identify suitable reference genes for analyzing functional gene expression patterns across different leaf colors and developmental stages of *L. formosan*.

#### 3.3.1. ΔCt Analysis

The stability ranking can be determined by the dispersion of gene Ct value data, with lower ΔCt values indicating higher expression stability [20]. Table 2 presents the ranking of the 14 candidate reference genes based on their expression stability in the leaf tissues of the 5 differently colored *L. formosan* leaves, calculated using the ΔCt method. The analysis revealed that *LifEF1-α* was the most stable reference gene in the September leaves, and *LifDnaJ* in the December leaves. Overall, *LifEF1-α* was identified as the ideal reference gene, followed by *LifACT*, whereas *LifLTP* was consistently deemed the least stable. The stability ranking of the reference genes across all samples was as follows: *LifEF1-α* > *LifACT* > *LifHIS* > *LifUBQ* > *LifSAND* > *LifH2A* > *LifDnaJ* > *Lifβ-TUB* > *LifGAPDH* > *Lifα-TUB* > *LifUBC* > *LifARF2* > *LifCDC2* > *LifLTP*. *LifEF1-α* emerged as an optimal reference gene.

#### 3.3.2. geNorm Analysis

**Figure 3:**
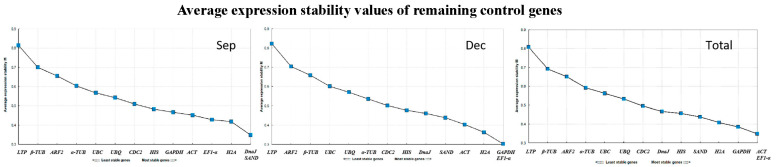
GeNorm analyzed the M values of the 14 candidate reference genes. We analyzed the M values of reference genes in leaves at different developmental stages and total samples using the geNorm algorithm and provided the optimal combination of reference genes.

Using the geNorm software, the M value was calculated, indicating the expression stability of each candidate reference gene across *L. formosan* leaf varieties. The software set M = 1.5 as the critical threshold, with lower M values indicating a higher stability. The analysis revealed that all 14 candidate reference genes had M values below 1.5, indicating stable and robust expression (Figure 3). Notably, *LifDnaJ*, *LifSAND*, *LifH2A*, *LifEF1-α*, *LifACT*, *LifGAPDH*, and *LifHIS* exhibited M values below 0.5 across all leaf samples, indicating a highly stable expression. Specifically, *LifDnaJ* and *LifSAND* demonstrated the highest expression stability in September leaves, whereas *LifGAPDH* and *LifEF1-α* demonstrated the highest stability in December leaves. Overall, *LifACT* and *LifEF1-α* were identified as the two reference genes with the highest expression stabilities across all leaf samples. These results indicated the variability in reference gene stability across the different developmental stages of the leaves.

**Figure 4:**
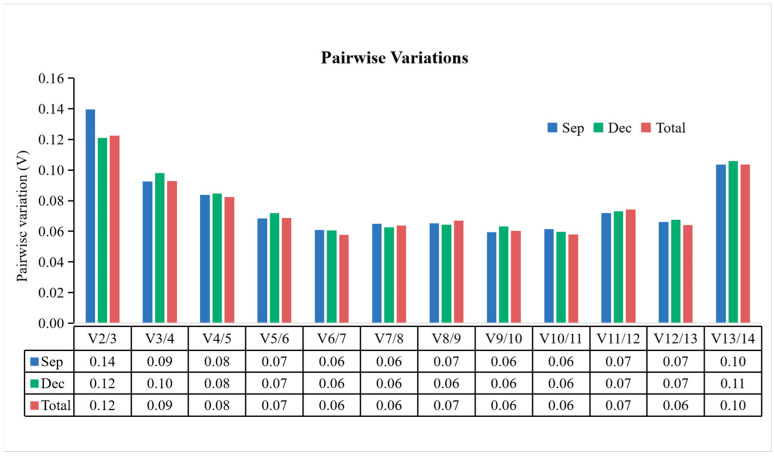
GeNorm assessed the ideal quantity of the reference genes by conducting paired variation analysis (V_n/n+1_). The threshold value for V_n/n+1_ was set at 0.15, with n denoting the optimal number of reference genes.

Furthermore, the geNorm software has the capability to conduct paired difference (V_n/n+1_) analysis on standardized factors of candidate reference genes to determine the optimal number of reference genes. When V_n/n+1_ < 0.15, n reference genes are sufficiently stable for normalization, obviating the need to introduce the n + 1st reference gene. This study revealed that V2/3 was less than 0.15 when analyzing leaf tissues separately in September and December, as well as when analyzing all samples collectively (Figure 4), indicating that a combination of two stable genes could improve the reliability and accuracy of the quantitative results. Overall, the comprehensive analysis ranked the stability of the 14 reference genes as follows: *LifACT = LifEF1-α* > *LifGAPDH* > *LifH2A* > *LifSAND* > *LifHIS* > *LifDnaJ* > *LifCDC2* > *LifUBC* > *LifUBQ* > *Lifα-TUB* > *LifARF2* > *Lifβ-TUB* > *LifLTP*, and *LifACT* and *LifEF1-α* emerged as the most suitable reference gene combination.

#### 3.3.3. BestKeeper Analysis

The BestKeeper primarily assesses gene stability by analyzing the standard deviation (SD) and coefficient of variation (CV) of the Ct values of candidate reference genes [21]. Smaller SD and CV values indicate greater stability, with an SD value of less than 1.0, which is considered indicative of high stability. In both the total sample and September sample, six reference genes had SD values < 1.0, whereas in the December sample, only four reference genes met this criterion (Table 3). The *LifEF1-α* consistently displayed SD values of <1.0 across all analyses, indicating the highest expression stability. The *LifACT* ranked second in expression quantity in September and December samples and third in the comprehensive analysis. Based on the SD and CV of the Ct values, reference gene stability was ranked as follows: *LifEF1-α* > *LifSAND* > *LifACT* > *Lifβ-TUB* > *LifDnaJ* > *LifHIS* > *LifUBQ* > *LifH2A* > *LifLTP* > *Lifα-TUB* > *LifGAPDH* > *LifUBC* > *LifCDC2* > *LifARF2* (Table 3).

#### 3.3.4. NormFinder Analysis

The NormFinder software evaluates the gene stability based on the stable expression value (S value). Smaller S values indicate greater stability. Subsequently, the optimal reference gene is selected [22]. In this study, the stability of the 14 reference genes across 5 *L. formosan* leaf slices with distinct color differences was analyzed. The results revealed the variations in gene stability among different samples. The *LifEF1-α* was deemed the most stable gene expression in the September samples, while the *LifDnaJ* demonstrated the highest stability in the December samples. Overall, the *LifACT* demonstrated the most stable expression when all samples were analyzed comprehensively. Conversely, the *LifLTP* exhibited the highest S value across all the analyses, indicating that it was the most unstable reference gene. The expression stability of the reference genes in all *L. formosan* leaves, ranked from high to low, was as follows: *LifACT* > *LifEF1-α* > *LifSAND* > *LifUBQ* > *LifHIS* > *LifH2A* > *LifDnaJ* > *Lifβ-TUB* > *LifGAPDH* > *Lifα-TUB* > *LifUBC* > *LifARF2* > *LifCDC2* > *LifLTP* (Table 4).

#### 3.3.5. RefFinder Analysis

The stability of the 14 candidate reference genes in *L. formosan* leaves at different developmental stages varied owing to the applications of different algorithms across software, in both within- and between-group analyses. To mitigate potential errors stemming from individual reference gene evaluation programs, we employed the RefFinder tool to comprehensively rank the results from the four aforementioned software packages. RefFinder utilized a comprehensive approach to analyze gene expression stability based on the geometric average weights of the four algorithms (GeNorm, NormFinder, ΔCt, and BestKeeper). Lower geometric average weight values indicate higher expression stability. RefFinder provided a more authoritative evaluation of the stability of the 14 candidate reference genes, yielding a more reasonable ranking. The overall stability ranking, from high to low, was as follows: *LifEF1-α* > *LifACT* > *LifSAND* > *LifUBQ* > *LifHIS* > *LifH2A* > *LifDnaJ* > *Lifβ-TUB* > *LifGAPDH* > *Lifα-TUB* > *LifUBC* > *LifARF2* > *LifCDC2* > *LifLTP*, suggesting that *LifEF1-α* and *LifACT* were the optimal reference gene combinations (Table 5). These two genes consistently ranked in the top five across the four algorithms, making them suitable reference genes for analyzing functional color gene expression patterns in *L. formosan* leaves. Additionally, the comprehensive analysis identified *LifCDC2* and *LifLTP* as the two most unstable genes that were unsuitable for use as reference genes.

### 3.4. Reference Gene Stability Verification

**Figure 5:**
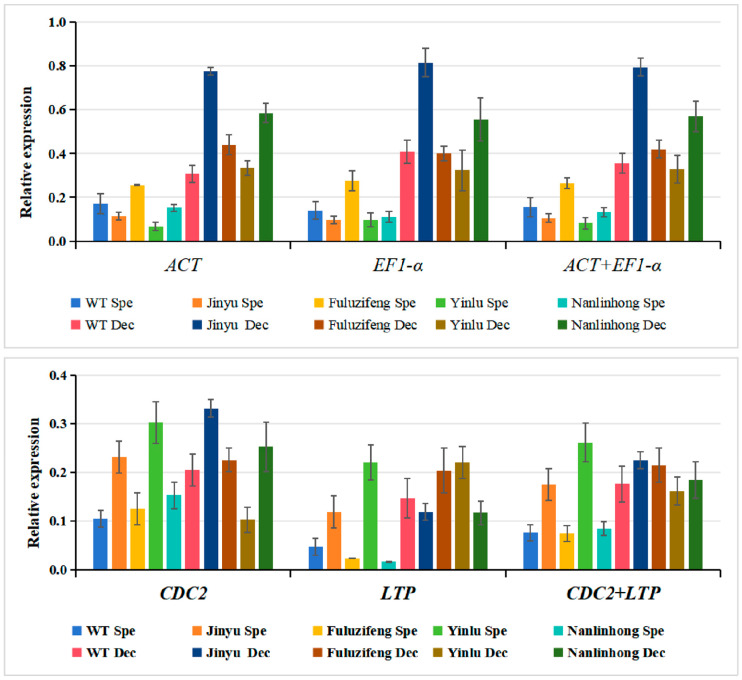
Verifying the stability of selected maple reference genes with *bHLH137*. Using RT-qPCR to evaluate the expression patterns of *bHLH137* in different leaf colors of maple leaves, normalizing gene expression data with *LifEF1-α*, *LifACT*, or a combination of both, and using *LifCDC2* and *LifLTP* as controls with the least stable expression. Analyzing gene expression through the 2^−ΔΔCt^ method.

The red and purple hues of *L. formosan* leaves stem from their high anthocyanin content, with cyanidin and paeoniflorin contributing to the red coloration, whereas delphinidin and geranium accumulation can enhance the purple appearance [3]. Previous investigations employed transcriptomics and metabolomics to jointly analyze the anthocyanin synthesis pathways in WT, “Jinyu”, “Fuluzifeng”, “Yinlu”, and “Nanlinhong”. The 14 candidate reference genes were stably expressed in leaves at different developmental stages (Figure S4A). The *bHLH137*, which promotes anthocyanin biosynthesis, was markedly upregulated during the leaf reddening (Figure S4B). The expression patterns of *bHLH137* across different *L. formosan* varieties were examined using RT-qPCR. The *LifEF1-α* and *LifACT*, selected as optimal internal reference genes, along with their combination, *LifEF1-α* + *LifACT*, were employed for normalization. Both individual and combined corrections yielded consistent relative expression levels of the target genes. The results indicated significant seasonal variation in the *bHLH137* expression across various varieties, with notable upregulation observed in the red autumn leaves. Especially in “Jinyu”, notable seasonal variation suggested that it may play a pivotal role in regulating leaf color changes. However, when utilizing the unstable expression of *LifCDC2* and *LifLTP* genes as references, the *bHLH137* expression patterns varied, lacking clear consistency (Figure 5). Inappropriate internal reference genes can lead to deviations in target gene expression levels, thereby compromising experimental accuracy. This reaffirmed the accuracy and reliability of the selected reference genes, *LifEF1-α* and *LifACT*, laying a foundation for future research on functional gene expression analysis related to seasonal shifts in *L. formosan* leaf color.

## 4. Discussion

*L. formosan*, a relic plant dating back to the third century, thrives in subtropical and tropical regions and displays diverse leaf colors, indicative of its adaptability to climate fluctuations [27]. Recently, there has been increased research interest in *L. formosan*, owing to its vibrant foliage and growing landscape value [8]. However, the lack of a comprehensive genome database has hindered reference gene screening in *L. formosan*, which is critical for analyzing functional gene expression patterns. Consequently, the depth of understanding of the mechanism of leaf color change in *L. formosan* remains limited. Although the *MYB113* has been implicated in leaf color regulation, its scope is primarily limited to autumn leaves and aging factors [28]. Research on candidate transcription factors that are directly involved in seasonal leaf color regulation is scarce. To elucidate the molecular mechanisms underlying leaf color variation across seasons or varieties of *L. formosan*, stable reference genes are essential for data standardization. Considering the limited development of reference genes in woody plants, it is imperative to screen for stable reference gene expression in diverse *L. formosan* varieties to lay the groundwork for subsequent research on related functional genes.

This study employed the RT-qPCR to comprehensively assess the expression stability of 14 candidate reference genes across 5 maple trees exhibiting significant leaf color variations [29]. Currently, multiple software tools are used for the RT-qPCR reference primer screening and evaluation. However, varying the calculation principles of these software programs can lead to contradictory conclusions from the same data. For instance, in a reference gene screening study of *Solanum melongena*, using only two software tools (geNorm and NormFinder) resulted in inconsistent results [30]. Conversely, in a study of *Chrysanthemum lavandulifolium* reference genes, the utilization of at least three software tools improved reliability [31]. Therefore, in this study, four software tools, including the geNorm version 2003, NormFinder version 0953, ΔCt version 2003, and BestKeeper version 2003, were employed for reference gene screening in *L. formosan*. The analysis results from the ΔCt and BestKeeper indicated *LifEF1-α* as the most stable reference gene across all samples, whereas the NormFinder identified *LifACT* as the most stable reference gene. Despite variations in the results from different software analyses, the ranking of candidate reference genes based on expression stability remained consistent across all samples. The geNorm software conducted a paired difference analysis of standardized factors and determined two reference genes as optimal. To mitigate errors arising from disparate algorithms, the RefFinder, a comprehensive analysis software, was applied to calculate the geometric mean of the four software analysis results. Ultimately, *LifEF1-α* and *LifACT* were identified as optimal reference gene combinations for standardized fluorescence-quantitative analysis in *L. formosan*.

The reference genes identified in this study provide an urgently needed resource for analyzing the expression patterns of functional genes in different varieties of *L. formosana* leaves in the future. Our study did not identify an independent gene that could serve as a suitable reference gene in *L. formosana* leaves of varying colors. This may be attributed to variations in experimental methodologies, materials, and the type and quantity of selected reference genes, all of which can influence the stability of reference gene expression. A single reference gene may lead to inaccurate quantification of target genes, and selecting a combination of reference genes for normalization is consistent with previous studies. Through comprehensive stability ranking in *Solanum lycopersicum*, *ACT* + *TUB* was selected as the best reference gene combination for leaves, and TUB + UVR8 was selected as the best reference gene combination for roots [32]. Similarly, in *Salix suchowensis*, comprehensive analysis using five software programs (Delta Ct, BestKeeper, NormFinder, GeNorm, and RefFinder) provided stable reference gene combinations *ACT* and *DnaJ* for male and female flowers [33]. Therefore, different statistical calculations should be used to determine the most suitable reference gene for different experimental conditions. On the basis of the stability results, combinations of reference genes are recommended for use in RT-qPCR.

This study identified *LifEF1-α* and *LifACT* as reference genes for variously colored *L. formosana* leaves. This selection facilitates the analysis of gene expression associated with color changes in different *L. formosana* leaves and establishes a foundation for breeding new *L. formosana* varieties with diverse leaf colors through advanced scientific and technological methods. Additionally, it offers a reference framework for the selection of reference genes in related species of *L. formosana*, such as *L. aclycina* Chang, *L. styraciflua* L., and *L. orientalis* Mill. Although homologous reference genes typically exhibited conservatism among closely related species, the variations in expression stability between species were also evident. Therefore, a comprehensive analysis involving multiple reference gene types could be essential for accurately evaluating target gene expression and quantitative analysis results across different species.

## 5. Conclusions

In this study, we used RT-qPCR to analyze the expression of 14 candidate reference genes in *L. formosana*, and then analyzed their stability using 5 different statistical algorithms. We concluded that *LifEF1-α* and *LifACT* were the most suitable RT-qPCR reference genes for *L. formosana* leaves with different leaf colors, and *LifbHLH137* was used as a target gene to validate the stability of the selected reference genes. To the best of our knowledge, this is the first study to identify the suitable reference genes for normalizing the gene expression studies using RT-qPCR in *L. formosana*. This lays the foundation for gene expression and other related molecular biology research on *L. formosana*.

## Figures and Tables

**Table 1:** Primer sequences and amplification efficiency of the 14 candidate reference genes and 1 target gene in *L. formosan*.

Gene	Gene Description	Primer Sequence F/R (5′-3′)	Product Size (bp)	Efficiency (%)	R^2^
*α-TUB1*	Alpha-tubulin 1	AGCAACTCATCAGCGGCAAGG	264	101.21	0.9801
		TGGCTCCACAACAGAGGTAGAAA			
*β-TUB*	Tubulin	ACGAGGCTCTTTATGATATTTG	264	107.22	0.9960
		GTTGGGTGAGTTCAGGGACA			
*ACT*	Actin	ATGTTCCCTGGCATTGCAGAC	181	102.45	0.9916
		ACTCATCATATTCACCCTTCG			
*ARF2*	ADP-ribosylation factor 2	GTTGAATGAGGATGAGTTGAGGGAA	177	105.14	0.9813
		CGTTGACGGAGCGAGTGAAGA			
*CDC2*	Cyclin-dependent kinase-putative	TGGCATTGCTTATTGTCATTC	123	106.40	0.9875
		GGTGCTCTGTACCACAGGGTC			
*DnaJ*	Chaperone protein DnaJ 49	TGGGTCAGATGAGCCCGTTTA	135	105.27	0.9923
		AATTGGGTCGGTTGCATTCCT			
*EF1-α*	Elongation factor 1-alpha	GATTGGTGGCATTGGAACTGT	124	109.53	0.9903
		GTGGTGCATCTCAACGGACTT			
*GAPDH*	Glyceraldehyde-3-phosphate dehydrogenase	GGTGGATTTGGCACATTTGGT	108	105.90	0.9944
		CACTCCTCATCAGCAGGGTTT			
*H2A*	Histone H2A	GCTTGAGTTGGCGGGAAATGC	168	103.12	0.9961
		TTCGGAAGGAGAAGGTTGTGAATGT			
*HIS*	Histone superfamily protein H3	GAAGAAGCCTCACAGATACCG	135	105.02	0.9950
		GTCTTGAAGTCCTGGGCAAT			
*LTP*	Seed storage/lipid transfer protein	CATCAGCAGCACAAGATTCAA	102	117.49	0.9765
		AAAGCATAACAGCACAGAGGC			
*SAND*	SAND family protein	GGCTTCAGAGTTTCCATCACC	157	107.50	0.9843
		GACCCAGCAGAGTAGAACATAG			
*UBC*	Ubiquitin conjugating enzyme	AGCCCTGCCCTTACCATTTCC	141	108.51	0.9920
		GCTCCTTGCGGTTGTTTCATA			
*UBQ*	Ubiquitin family	AGGAGTGCCCTAATGCCGAGTG	105	99.31	0.9947
		CAGCCTTCTGATAAACGTAAGTCAA			
Target gene					
*bHLH137*	Basic helix-loop-helix	AAGAAGCTCCAACAGGGTAC	200	101.77	0.9972
		TTGTAGGGACTGGACATAGTT			

**Table 2:** ΔCt analysis of the average SD of the 14 candidate reference genes in different samples of *L. formosan*.

Rank		1	2	3	4	5	6	7	8	9	10	11	12	13	14
Spe	Gene	*EF1-α*	*DnaJ*	*SAND*	*H2A*	*CDC2*	*α-TUB*	*UBQ*	*HIS*	*UBC*	*β-TUB*	*ACT*	*GAPDH*	*ARF2*	*LTP*
	Stability	0.63	0.65	0.67	0.68	0.69	0.74	0.75	0.76	0.78	0.8	0.92	1.02	1.1	1.45
Dec	Gene	*DnaJ*	*EF1-α*	*SAND*	*HIS*	*β-TUB*	*H2A*	*CDC2*	*UBQ*	*α-TUB*	*UBC*	*ACT*	*ARF2*	*GAPDH*	*LTP*
	Stability	0.6	0.64	0.64	0.65	0.66	0.66	0.69	0.75	0.77	0.79	0.82	0.96	1.03	1.6
Total	Gene	*EF1-α*	*ACT*	*HIS*	*UBQ*	*SAND*	*H2A*	*DnaJ*	*β-TUB*	*GAPDH*	*α-TUB*	*UBC*	*ARF2*	*CDC2*	*LTP*
	Stability	0.41	0.42	0.44	0.46	0.51	0.58	0.69	0.87	0.93	1.04	1.25	1.68	2.58	2.67

**Table 3:** BestKeeper software analysis of the expression stability of the candidate reference genes.

Rank		1	2	3	4	5	6	7	8	9	10	11	12	13	14
Spe	Gene	*EF1-α*	*ACT*	*β-TUB*	*DnaJ*	*UBQ*	*HIS*	*LTP*	*SAND*	*α-TUB*	*H2A*	*CDC2*	*UBC*	*GAPDH*	*ARF2*
	SD	0.69	0.76	0.78	0.86	0.91	0.92	1.08	1.19	1.27	1.34	1.51	1.57	1.71	1.93
	CV	2.07	2.36	2.52	3.21	3.41	3.47	3.88	4.87	4.98	5.37	5.62	6.02	7.03	8.41
Dec	Gene	*EF1-α*	*ACT*	*DnaJ*	*β-TUB*	*α-TUB*	*UBQ*	*SAND*	*HIS*	*CDC2*	*H2A*	*LTP*	*UBC*	*ARF2*	*GAPDH*
	SD	0.61	0.64	0.72	0.81	2.47	2.55	2.58	2.64	2.76	2.89	3.05	3.09	3.32	3.33
	CV	5.81	8.55	8.86	9.43	11.49	11.69	9.94	11.65	10.88	11.61	12.42	13.29	13.61	14.12
Total	Gene	*EF1-α*	*SAND*	*ACT*	*β-TUB*	*DnaJ*	*HIS*	*UBQ*	*H2A*	*LTP*	*α-TUB*	*GAPDH*	*UBC*	*CDC2*	*ARF2*
	SD	0.70	0.77	0.80	0.82	0.91	0.94	1.16	1.21	1.48	1.62	1.94	1.96	2.21	2.48
	CV	2.38	2.55	2.76	3.15	3.21	3.48	4.16	4.85	5.41	6.21	8.65	8.98	9.85	10.81

**Table 4:** NormFinder analysis of stable values of the 14 candidate reference genes in *L. formosan*.

Rank		1	2	3	4	5	6	7	8	9	10	11	12	13	14
Spe	Gene	*EF1-α*	*SAND*	*DnaJ*	*CDC2*	*H2A*	*α-TUB*	*HIS*	*UBQ*	*UBC*	*β-TUB*	*ACT*	*GAPDH*	*ARF2*	*LTP*
	Stability	0.158	0.249	0.267	0.34	0.34	0.425	0.467	0.496	0.537	0.544	0.74	0.899	0.988	1.381
Dec	Gene	*DnaJ*	*SAND*	*EF1-α*	*HIS*	*β-TUB*	*H2A*	*CDC2*	*α-TUB*	*UBQ*	*UBC*	*ACT*	*ARF2*	*GAPDH*	*LTP*
	Stability	0.114	0.242	0.264	0.285	0.292	0.329	0.387	0.525	0.527	0.583	0.588	0.84	0.941	1.560
Total	Gene	*ACT*	*EF1-α*	*SAND*	*UBQ*	*HIS*	*H2A*	*DnaJ*	*β-TUB*	*GAPDH*	*α-TUB*	*UBC*	*ARF2*	*CDC2*	*LTP*
	Stability	0.176	0.263	0.274	0.378	0.411	0.467	0.482	0.497	0.896	1.063	1.250	1.453	1.533	1.874

**Table 5:** Comprehensive analysis results of RefFinder.

Rank	1	2	3	4	5	6	7	8	9	10	11	12	13	14
Ranking order of leaves in September (better average)														
Delta Ct	*EF1-α*	*DnaJ*	*SAND*	*H2A*	*CDC2*	*α-TUB*	*UBQ*	*HIS*	*UBC*	*β-TUB*	*ACT*	*GAPDH*	*ARF2*	*LTP*
geNorm	*DnaJ*	*SAND*	*H2A*	*EF1-α*	*ACT*	*GAPDH*	*HIS*	*CDC2*	*UBQ*	*UBC*	*α-TUB*	*ARF2*	*β-TUB*	*LTP*
BestKeeper	*EF1-α*	*ACT*	*β-TUB*	*DnaJ*	*UBQ*	*HIS*	*LTP*	*SAND*	*α-TUB*	*H2A*	*CDC2*	*UBC*	*GAPDH*	*ARF2*
NormFinder	*EF1-α*	*SAND*	*DnaJ*	*CDC2*	*H2A*	*α-TUB*	*HIS*	*UBQ*	*UBC*	*β-TUB*	*ACT*	*GAPDH*	*ARF2*	*LTP*
Comprehensive	*EF1-α*	*SAND*	*DnaJ*	*H2A*	*CDC2*	*α-TUB*	*HIS*	*UBQ*	*UBC*	*β-TUB*	*ACT*	*GAPDH*	*ARF2*	*LTP*
Ranking order of leaves in December (better average)														
Delta Ct	*DnaJ*	*EF1-α*	*SAND*	*HIS*	*β-TUB*	*H2A*	*CDC2*	*UBQ*	*α-TUB*	*UBC*	*ACT*	*ARF2*	*GAPDH*	*LTP*
geNorm	*GAPDH*	*EF1-α*	*H2A*	*ACT*	*SAND*	*DnaJ*	*HIS*	*CDC2*	*α-TUB*	*UBQ*	*UBC*	*β-TUB*	*ARF2*	*LTP*
BestKeeper	*EF1-α*	*ACT*	*DnaJ*	*β-TUB*	*α-TUB*	*UBQ*	*SAND*	*HIS*	*CDC2*	*H2A*	*LTP*	*UBC*	*ARF2*	*GAPDH*
NormFinder	*DnaJ*	*SAND*	*EF1-α*	*HIS*	*β-TUB*	*H2A*	*CDC2*	*α-TUB*	*UBQ*	*UBC*	*ACT*	*ARF2*	*GAPDH*	*LTP*
Comprehensive	*DnaJ*	*EF1-α*	*ACT*	*SAND*	*β-TUB*	*H2A*	*CDC2*	*HIS*	*α-TUB*	*UBQ*	*UBC*	*ARF2*	*GAPDH*	*LTP*
Ranking order under total samples (better average)														
Delta Ct	*EF1-α*	*ACT*	*HIS*	*UBQ*	*SAND*	*H2A*	*DnaJ*	*β-TUB*	*GAPDH*	*α-TUB*	*UBC*	*ARF2*	*CDC2*	*LTP*
geNorm	*ACT*	*EF1-α*	*GAPDH*	*H2A*	*SAND*	*HIS*	*DnaJ*	*CDC2*	*UBQ*	*UBC*	*α-TUB*	*ARF2*	*β-TUB*	*LTP*
BestKeeper	*EF1-α*	*SAND*	*ACT*	*β-TUB*	*DnaJ*	*HIS*	*UBQ*	*H2A*	*LTP*	*α-TUB*	*GAPDH*	*UBC*	*CDC2*	*ARF2*
NormFinder	*ACT*	*EF1-α*	*SAND*	*UBQ*	*HIS*	*H2A*	*DnaJ*	*β-TUB*	*GAPDH*	*α-TUB*	*UBC*	*ARF2*	*CDC2*	*LTP*
Comprehensive	*EF1-α*	*ACT*	*SAND*	*UBQ*	*HIS*	*H2A*	*DnaJ*	*β-TUB*	*GAPDH*	*α-TUB*	*UBC*	*ARF2*	*CDC2*	*LTP*

## Data Availability

Data are contained within the article and Supplementary Materials.

## Supplementary Materials

The following supporting information can be downloaded at: https://www.mdpi.com/article/10.3390/cimb46090560/s1, Figure S1: 1% agarose gel electrophoresis shows specific bands of PCR products. Figure S2: Melting curves of the 14 reference genes showing single peaks. Figure S3: Amplification plots of the 14 candidate reference genes. Figure S4: Expression profiles of the 14 candidate reference genes and the *bHLH137* gene in different samples.
