# Supplementary material for: Selection and Validation of Reliable Reference Genes for Liquidambar formosana Leaves with Different Leaf Colors
Source: Curr Issues Mol Biol. 2024 Aug 27;46(9):9449–62. doi: 10.3390/cimb46090560 (PMC11430312; doi:10.3390/cimb46090560)
Supplement: Supplementary file 1 [file cimb-46-00560-s001.zip › cimb-3142840-supplementary.pdf]

Supplementary Materials

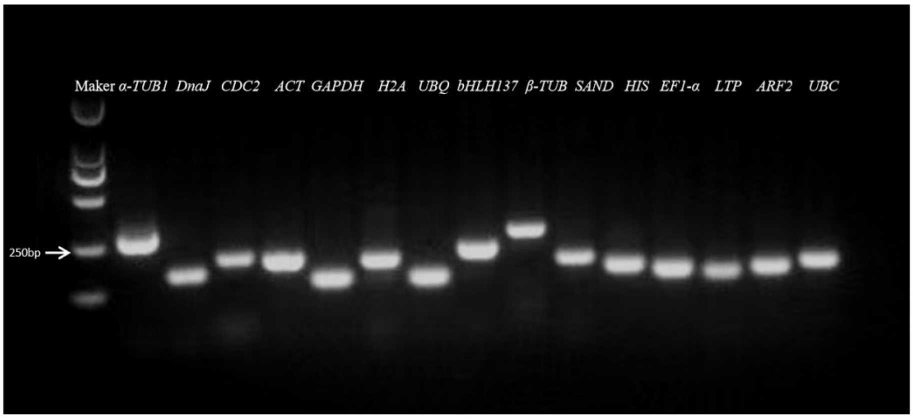

Figure S1. 1% agarose gel electrophoresis shows specific bands of PCR products.

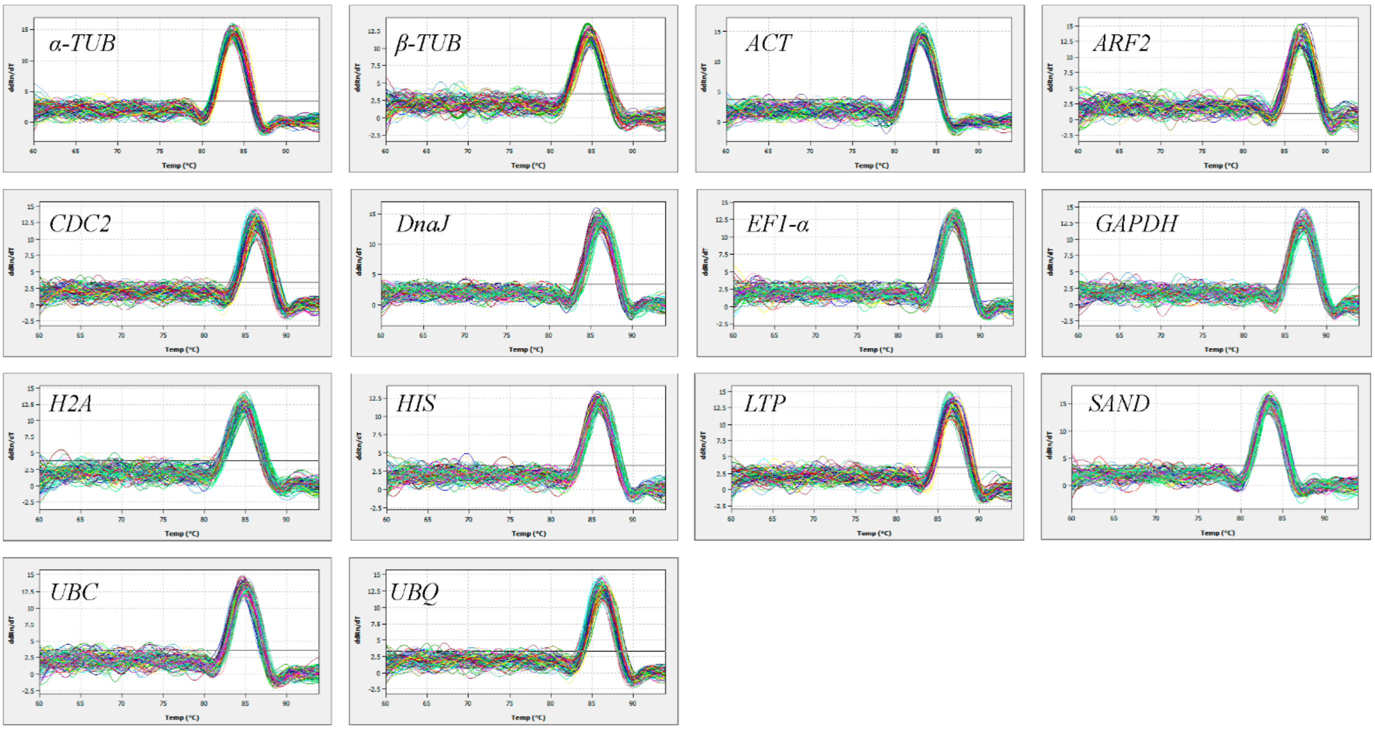

Figure S2. Melting curves of 14 reference genes showing single peaks.

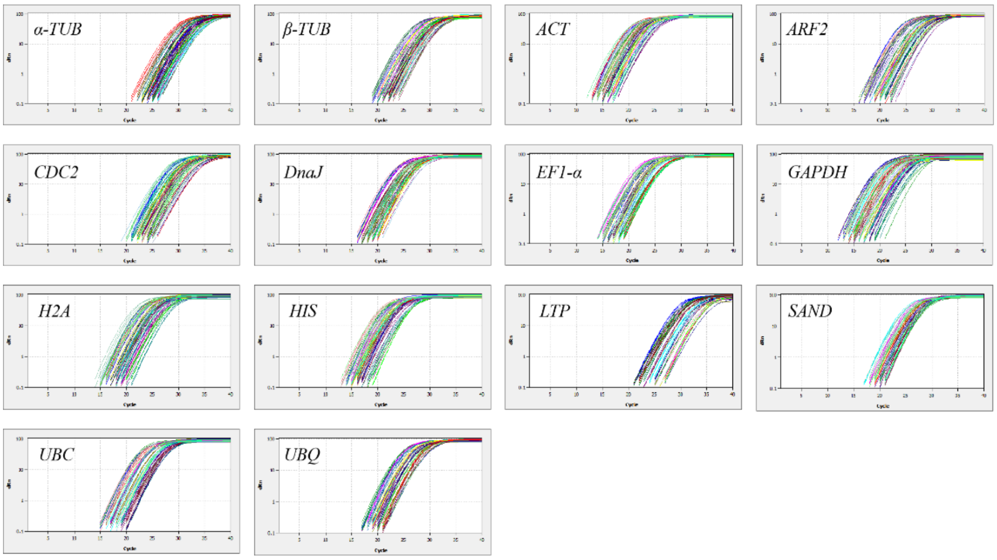

Figure S3. Amplification plots of the 14 candidate reference genes.

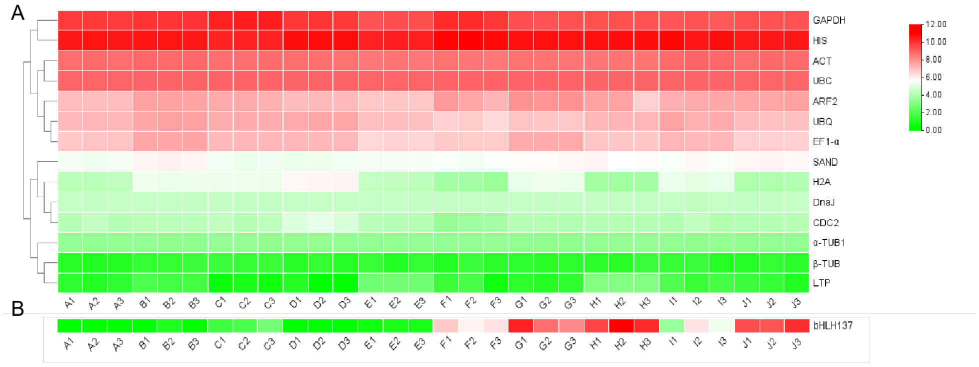

Figure S4. Expression profiles of 14 candidate reference genes and *bHLH137* gene in different samples.
